# The Protein Identifier Cross-Referencing (PICR) service: reconciling protein identifiers across multiple source databases

**DOI:** 10.1186/1471-2105-8-401

**Published:** 2007-10-18

**Authors:** Richard G Côté, Philip Jones, Lennart Martens, Samuel Kerrien, Florian Reisinger, Quan Lin, Rasko Leinonen, Rolf Apweiler, Henning Hermjakob

**Affiliations:** 1 European Bioinformatics Institute, Wellcome Trust Genome Campus, Hinxton, Cambridge, CB10 1QY, UK

## Abstract

**Background:**

Each major protein database uses its own conventions when assigning protein identifiers. Resolving the various, potentially unstable, identifiers that refer to identical proteins is a major challenge. This is a common problem when attempting to unify datasets that have been annotated with proteins from multiple data sources or querying data providers with one flavour of protein identifiers when the source database uses another. Partial solutions for protein identifier mapping exist but they are limited to specific species or techniques and to a very small number of databases. As a result, we have not found a solution that is generic enough and broad enough in mapping scope to suit our needs.

**Results:**

We have created the Protein Identifier Cross-Reference (PICR) service, a web application that provides interactive and programmatic (SOAP and REST) access to a mapping algorithm that uses the UniProt Archive (UniParc) as a data warehouse to offer protein cross-references based on 100% sequence identity to proteins from over 70 distinct source databases loaded into UniParc. Mappings can be limited by source database, taxonomic ID and activity status in the source database. Users can copy/paste or upload files containing protein identifiers or sequences in FASTA format to obtain mappings using the interactive interface. Search results can be viewed in simple or detailed HTML tables or downloaded as comma-separated values (CSV) or Microsoft Excel (XLS) files suitable for use in a local database or a spreadsheet. Alternatively, a SOAP interface is available to integrate PICR functionality in other applications, as is a lightweight REST interface.

**Conclusion:**

We offer a publicly available service that can interactively map protein identifiers and protein sequences to the majority of commonly used protein databases. Programmatic access is available through a standards-compliant SOAP interface or a lightweight REST interface. The PICR interface, documentation and code examples are available at .

## Background

Biological data is being generated at an unparalleled rate and data analysis is becoming a key challenge in bioinformatics and systems biology. Two common tasks that are more difficult than they should be are identifier unification, where datasets from various sources must be merged together for analysis and identifier translation, where identifiers from one source (e.g. NCBI gi number) need to be converted to those from another source (e.g. Ensembl) so that they can be used in database specific tools and queries. A major hindrance to the effective implementation of those tasks is that data comes from multiple sources, each using a proprietary identifier scheme that is not always easily traceable to a specific provider.

It is common to observe the same protein sequence being referred to by multiple identifiers. Redundant databases may even assign multiple identifiers to the same sequence. This problem is compounded by the fact that identifiers are unstable and can (and do!) disappear from source databases. For example, it is common for hypothetical proteins to be replaced when gene prediction algorithms are updated. Identifiers from in-house or proprietary databases are unknown to the outside world. At best, protein identifier translation into a common search space is a tedious task. At worst, it is an impossible one.

The major reference databases, such as the Universal Protein Knowledge Base (UniProtKB) [1], Ensembl [2] and the NCBI RefSeq [3] maintain a comprehensive list of cross-references to each other but full coverage is difficult to achieve because these databases have different production cycle and release schedules. Smaller, more specialized databases or proprietary ones might not be included in the cross-referencing process described above and will not be linked from these databases. Ultimately, this means that users must still query multiple sources to ensure that they have a complete picture with the latest information available.

The mapping problem has been tackled before by many groups using varied approaches. Unified identifier schemes have been proposed in the past, such as Life Science Identifiers (LSID) [4] and Sequence Globally Unique Identifiers (SEGUID) [5], but their adoption remains limited.

Many tools have been investigated but were found wanting, either because of the limited scope of databases or species they cover, their lack of API to use for batch or programmatic access, or because they are slanted to use in one particular field. Others have limited usability, such as few variables per request or requiring knowledge about the exact source and destination database.

For example, SeqDB [6] imports sequence information from external sources and generates a list of known aliases. However, coverage of synonyms is only limited to a small number of source databases and is only available to use interactively online using a web browser. IDConverter and IDClight [7] are web-based tools that map between clones, gene identifiers and protein accession numbers but the mappings are restricted to three species (human, rat and mouse) and only cover a small number of sources. IDClight does offer the possibility to use web links to perform one mapping per request, but datasets are only refreshed every two months [8]. The National Cancer Institute caBIG GeneConnect project will offer both programmatic and interactive queries, but is currently limited to mappings between Ensembl, RefSeq and UniProt [9].

The ID Mapping service offered by Protein Information Resource (PIR) [10] has limited functionality in that it can only map between two sources per request, meaning that if the user wishes to map proteins from SGD, IPI and Genbank to UniProt, three requests must be made (SGD to UniProt, IPI to UniProt and Genbank to UniProt). Also, not all mappings are available. For example, it is possible to map from SGD to UniProt and from Genbank to UniProt, but not from SGD to Genbank.

MatchMiner [11] is aimed more towards gene name and gene product mappings and is limited to only two species (human and mouse). Onto-Translate [12], SOURCE [13] and Resourcerer [14] are designed to be used primarily for microarray and gene expression data analysis and as such, are not suitable for general use as they are gene-centric rather than protein-centric.

PROMPT [15] is a standalone comparative proteomics tool that can perform protein mapping based on sequence similarity as one of its functions. However, it is up to the user to download the source files and load them into the application. Mapping coverage is therefore limited to those sources the user installs and data freshness is only ensured by how often the user refreshes the source files. Furthermore, although it does provide an API to integrate some functionality in other applications, it does require that a local installation be maintained.

Our goal in starting this project was to build a service that would meet the following requirements:

• the ability to map sequences as well as protein identifiers;

• identifiers could come from multiple sources in one request;

• identifiers could be mapped to multiple destination databases in one request;

• mappings could be done interactively as well as programmatically;

• mappings could be limited to specific taxon identifiers or across all species;

• mappings could handle identifiers deleted from source databases but still available in result sets and the scientific literature;

• mappings could be done against all primary protein data sources;

• mappings could be done against most other protein data sources.

The first users of this service will be the Proteomics Identifications Database (PRIDE) [16,17] and the IntAct Database [18], to simplify the task of mapping large scale proteomics and interaction experiments to a common reference system. However, by implementing the abovementioned requirements, we would provide the most powerful, comprehensive and versatile public service for mapping protein identifiers across different data sources to the scientific community at large.

## Implementation

### System architecture

**Figure 1:**
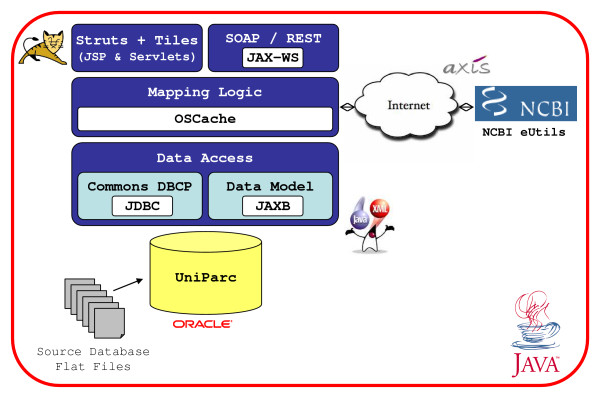
**PICR architecture**. PICR has a 3-tier architecture implemented in Java. The data access layer queries the UniParc database using a JDBC connection pool and provides model objects for the logic layer. The logic layer implements the mapping algorithm and uses SOAP to connect to the NCBI eUtils, as requested. The presentation layer has both interactive and service-oriented components, both hosted on a Tomcat server.

PICR is built using a classic 3-tier application model, as illustrated in Figure 1. The data layer is built around the UniProt Archive (UniParc). An in-depth description of UniParc and its production cycle can be found here [19]. The logic layer uses an API written in Java [20] to implement the mapping algorithm described below and return JAXB-annotated [21] data model objects to the presentation layer. The presentation layer uses Servlets and Java Server Pages (JSP) in the context of an Apache Struts [22] application. To make the application more responsive and provide a better browsing experience, AJAX is used wherever possible. The presentation layer also provides a JAX-WS [23] implemented SOAP service and a REST API.

To improve performance, database connection pooling (DBCP) is done using the Apache Commons DBCP [24] API at the data layer and caching is done where possible using the OpenSymphony Cache [25] API. Logging is done using Log4J [26] and real-time error reporting and user notification is done using the JavaMail [27] API.

### Data model

**Figure 2:**
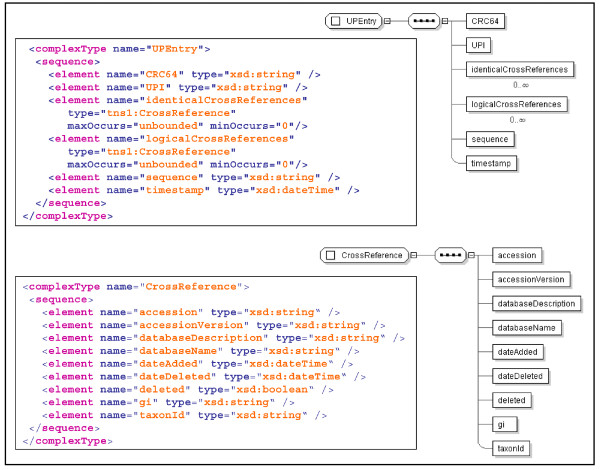
**XML schema for the PICR data model**. The XML Schema and modelled view of the PICR data model objects.

The data model for PICR is very simple and revolves around two objects: UPEntry and CrossReference. The XML schema of these objects is shown in Figure 2. UPEntry represents an entry in the UniParc database and will contain a protein sequence and its CRC64 checksum, a timestamp and two collections of CrossReference objects – one based on sequence identity and obtained from the XREF table of UniParc and one based on the data from UniProt. The meaning of each collection will be elaborated on in the explanation of the mapping algorithm, below.

CrossReference objects contain the description of the source database they originate from, the accession number and version of the entry, a status flag indicating if the entry is active (i.e. still available in the source database release files) or inactive (i.e. deleted from the source database), the date the entry was first loaded into UniParc as well as additional information such as the NEWT [28] taxonomy id (if available), the corresponding NCBI gi number (if available) and the date the entry was last loaded (if still active) or the date the entry was deleted (if such is the case).

## Results and discussion

**Figure 3:**
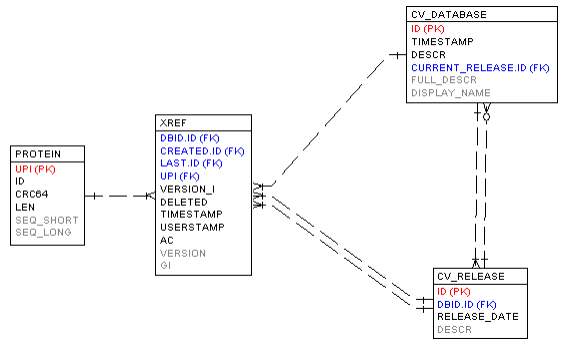
**Simplified UniParc database schema**. A simplified, partial view of the UniParc database schema that acts as the data warehouse for PICR data.

UniParc is the central data warehouse for PICR, though it can be complemented by external sources on occasion. The central tenet of UniParc is that each version of each sequence from each source database will be archived. Source databases are polled daily and updates are loaded into UniParc as soon as they become available. As such, UniParc is the largest and most comprehensive historical sequence archive available (Refer to statistics in Table 1). At time of writing, it contained 15.6 million distinct sequences loaded from 4,632 releases obtained from 73 distinct sources. This corresponds to 42.5 million non-unique protein identifiers and 37.6 unique protein identifiers. The disparity in the numbers is due to the nature of UniParc. As protein entries are updated, identifiers may be assigned to different protein sequences if the sequence associated with it has changed. Protein sequences are stored in the Protein table and are assigned a unique UniParc Protein Identifier (UPI) that will be invariant for the life of the protein sequence. As each source database is loaded in UniParc, if a protein sequence is already present, the source database identifier will be created or updated in the Xref table. If the protein sequence is novel, a new Protein entry will be created with an associated Xref entry (Figure 3).

**Table 1:** Data available in UniParc

**Source Name**	**Source Description**	**Number of Releases**	**Number of Entries**
EMBL	EMBL Nucleotide Sequence Database	883	4,776,027
EMBLWGS	Whole Genome Shotgun	256	2,894,683
EMBL_ANNCON	Annotated CON entries	63	6,773,092
EMBL_TPA	Third Party Annotation	74	5,497
ENSEMBL_ARMADILLO	Ensembl Dasypus novemcinctus	8	15,552
ENSEMBL_BUSHBABY	Ensembl Otolemur garnettii	3	15,449
ENSEMBL_CAT	Ensembl Felis catus	4	14,846
ENSEMBL_CBRIGGSAE	Ensembl Caenorhabditis briggsae	14	14,713
ENSEMBL_CELEGANS	Ensembl Caenorhabditis elegans	35	39,090
ENSEMBL_CHICKEN	Ensembl Gallus gallus	29	67,610
ENSEMBL_CHIMP	Ensembl Pan troglodytes	30	83,636
ENSEMBL_CIONA	Ensembl Ciona intestinalis	18	40,996
ENSEMBL_COMMON_SHREW	Ensembl Sorex araneus	2	13,195
ENSEMBL_COW	Ensembl Bos taurus	17	82,819
ENSEMBL_DOG	Ensembl Canis familiaris	22	52,106
ENSEMBL_ELEPHANT	Ensembl Loxodonta africana	8	15,717
ENSEMBL_ERINACEUS	Ensembl Erinaceus europaeus	4	14,593
ENSEMBL_FLY	Ensembl Drosophila melanogaster	35	25,934
ENSEMBL_FUGU	Ensembl Fugu rubripes	36	112,525
ENSEMBL_GUINEA_PIG	Ensembl Cavia porcellus	4	28,438
ENSEMBL_HEDGEHOG	Ensembl Echinops telfairi	8	16,582
ENSEMBL_HONEYBEE	Ensembl Apis mellifera	18	43,953
ENSEMBL_HUMAN	Ensembl Homo sapiens	35	115,689
ENSEMBL_MEDAKA	Ensembl Oryzias latipes	6	25,880
ENSEMBL_MICROBAT	Ensembl Myotis lucifugus	3	16,234
ENSEMBL_MOSQUITO	Ensembl Anopheles gambiae	35	55,270
ENSEMBL_MOUSE	Ensembl Mus musculus	37	127,637
ENSEMBL_OPOSSUM	Ensembl Monodelphis domestica	13	54,269
ENSEMBL_PLATYPUS	Ensembl Ornithorhynchus anatinus	5	32,001
ENSEMBL_RABBIT	Ensembl Oryctolagus cuniculus	8	15,441
ENSEMBL_RAT	Ensembl Rattus norvegicus	35	89,524
ENSEMBL_RHESUS_MACAQUE	Ensembl Macaca mulatta	11	61,299
ENSEMBL_SQUIRREL	Ensembl Spermophilus tridecemlineatus	3	14,833
ENSEMBL_STICKLEBACK	Ensembl Gasterosteus aculeatus	8	27,671
ENSEMBL_TETRAODON	Ensembl Tetraodon nigroviridis	27	28,004
ENSEMBL_TREE_SHREW	Ensembl Tupaia belangeri	4	15,462
ENSEMBL_XENOPUS	Ensembl Xenopus tropicalis	21	76,758
ENSEMBL_YF_MOSQUITO	Ensembl Aedes aegypti	8	16,789
ENSEMBL_ZEBRAFISH	Ensembl Danio rerio	37	161,469
EPO	European Patent Office	11	780,113
FLYBASE	FlyBase	3	18,549
H_INV	H-Invitational Database	25	864,262
IPI	International Protein Index	58	910,640
JPO	Japan Patent Office	15	404,695
PDB	Protein Data Bank	261	112,882
PIR	PIR-PSD	17	283,420
PIRARC	PIR-PSD archive	2	342,752
PRF	Protein Research Foundation	77	791,254
REFSEQ	RefSeq release + updates	847	5,598,926
REFSEQ_HUMAN	REFSEQ Homo sapiens	154	105,699
REFSEQ_MOUSE	REFSEQ Mus musculus	153	152,647
REFSEQ_RAT	REFSEQ Rattus norvegicus	151	97,753
REFSEQ_ZEBRAFISH	REFSEQ Danio rerio	141	63,183
SGD	SGD Protein	16	6,002
SWISSPROT	UniProtKB/Swiss-Prot	213	333,918
SWISSPROT_VARSPLIC	SWISS-PROT alternative splicing	132	38,756
TAIR_ARABIDOPSIS	TAIR Arabidopsis thaliana	5	33,914
TREMBL	UniProtKB/TrEMBL	118	5,877,814
TREMBL_VARSPLIC	TrEMBL alternative splicing	78	1,051
TROME_CE	TROME Caenorhabditis elegans	18	84,895
TROME_DM	TROME Drosophila melanogaster	20	116,588
TROME_HS	TROME Homo sapiens	25	1,180,511
TROME_MM	TROME Mus musculus	24	675,662
UNIMES	UniProt Metagenomic and Environmental Sequences	1	6,028,191
USPTO	US Patent and Trademark Office	14	724,428
VEGA_DOG	Vega Canis familiaris	1	50
VEGA_HUMAN	Vega Homo sapiens	7	58,931
VEGA_MOUSE	Vega Mus musculus	7	20,750
VEGA_ZEBRAFISH	Vega Danio rerio	8	13,293
WORMBASE	WormBase	65	30,438

Data sources warehoused in UniParc. The source name should be used when using the REST and SOAP interfaces. The number of releases indicates how many times the source files have been parsed and loaded into UniParc and includes incremental and full releases. The number of entries corresponds to the total number of protein entries parsed for all the releases. Note that UniParc is based on 100% sequence identity so one protein entry might be repeated multiple times as versions are updated. Replaced entries are simply marked as inactive, but are never deleted in order to provide archival coverage.

### Mapping algorithm

**Figure 4:**
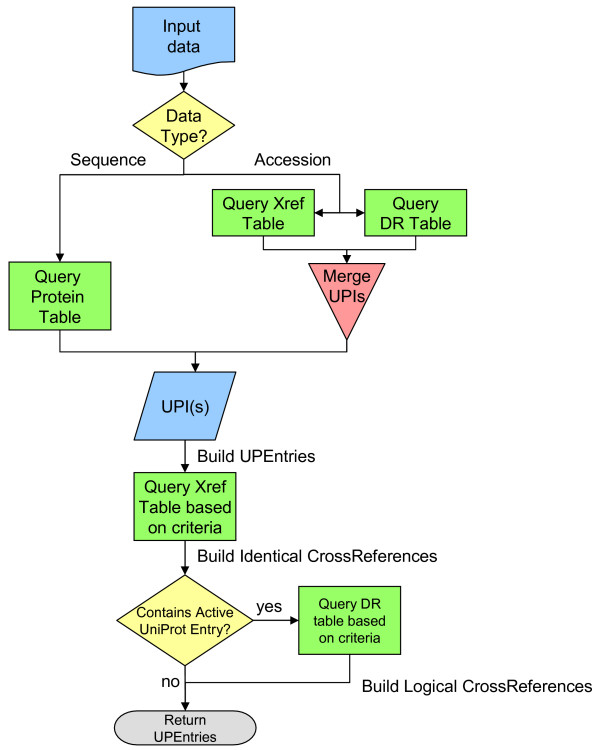
**Identifier mapping algorithm**. A flowchart view of the PICR mapping algorithm.

The complete mapping algorithm is illustrated in Figure 4 and has two phases. The first is to find the proper Protein entries that correspond to the input data, be it sequences or accessions. The second is to gather all known cross-references for each entry that fit the search criteria.

### Mapping by sequence

Once a sequence is submitted for mapping, a CRC64 checksum is computed for that sequence and is used to quickly and efficiently query the Protein table of UniParc. Mappings are done on the basis of 100% sequence identity over the whole sequence. Subsequence matches are not considered as valid mappings as they will not generate identical CRC64 values. If no entries are found, the sequence cannot be mapped. If multiple entries are found, due to checksum collisions, the sequences are retrieved from UniParc and only the matching one is kept. CRC64 collisions are very rare but will occur, given the sequence volume of UniParc. At time of writing, 0.000115% of the total number of sequences have CRC64 collisions.

A UPEntry object is created and the UPI, sequence and timestamps fields are populated. The UPI of the correctly identified sequence is used to retrieve the Xref entries associated with that sequence, based on the search criteria. These criteria include the selected databases to map to, the possibility to retrieve all mappings (including inactive or deleted cross-references) or only active ones and the possibility to limit mappings to a selected species. The entries obtained from the Xref table will then be used to create CrossReference objects and will be added to the IdenticalCrossReference collection of the UPEntry object as they are all based on 100% sequence identity.

If the submitted sequence happens to have an active UniProt (SwissProt or TREMBL) cross-reference, additional data is looked up in a separate table in the UniParc schema. This supplementary information table will contain additional information extracted from the current UniProt release files, including secondary identifiers, UniProt IDs (e.g. JAD1A_HUMAN for the protein whose accession number is P29375) and cross-references maintained by UniProt to data sources available in UniParc. These human-annotated (SwissProt) and automatically-derived (TREMBL) cross-references can provide added value as the mappings they provide, while valid, might be to sequences that are different to the main UniProt sequence (such as splice variants, sequencing errors, natural variations, etc). Such mappings would not normally have been available via UniParc unless the exact variant sequence was queried. However, since they may not represent the exact sequence, it was decided to keep them separated from those obtained based on sequence identity. As such, CrossReference objects created from those records are stored in the LogicalCrossReference collection of the UPEntry. Logical CrossReference data will also be filtered according to the search criteria (selected databases, activity status, taxonomy annotation).

Querying with taxonomy restrictions was designed to be pessimistic. While taxonomy annotation coverage is improving in UniParc, many databases do not provide taxonomy information. Xrefs entries that are not annotated with taxonomy information or are not an exact match to the query parameter will not be included in the search results.

### Mapping by accession

Mapping by protein identifier uses similar logic as that described above, but with a different starting point. If a protein accession is submitted, the supplementary information and Xref tables are queried to obtain all pertinent UPIs.

A UPEntry is created for each UPI and the relevant fields are populated from data gathered in the Protein table. The CrossReference collections of each UPI are then populated using the mechanisms described above. If a NCBI gi number is submitted (gi|1710032), the Xref table is queried as a starting point. However, gi number coverage is still low with respect to the overall number of entries in UniParc at only 41.5% at time of writing. If a gi number is not in UniParc, PICR will query the NCBI eUtilities [29] to obtain the corresponding sequence and use that as a starting point for mapping by sequence, as described above.

### Using PICR to map PRIDE identifications

**Figure 5:**
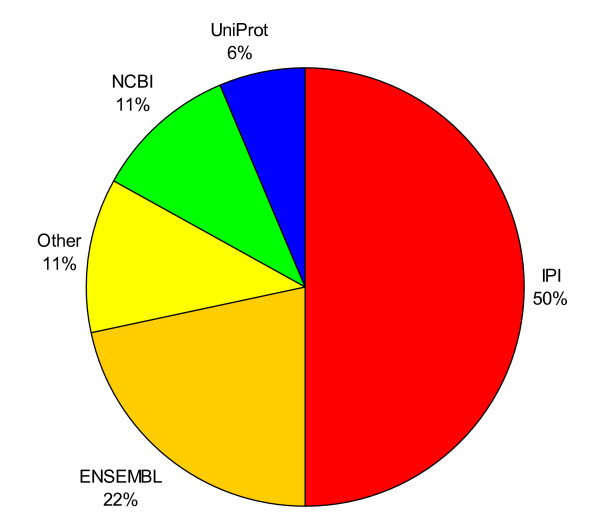
**Data sources for PRIDE identifications**. 89% of all PRIDE identifications are annotated with protein identifiers coming from IPI, UniProt, NCBI or Ensembl. NCBI entries are NR Accessions, RefSeq accessions or gi numbers. The rest come from more specialized or proprietary databases.

PRIDE is a user-driven submission database and will be a significant user of PICR. At time of writing, the distribution of data sources that were used to generate PRIDE identifications is shown in Figure 5.

**Figure 6:**
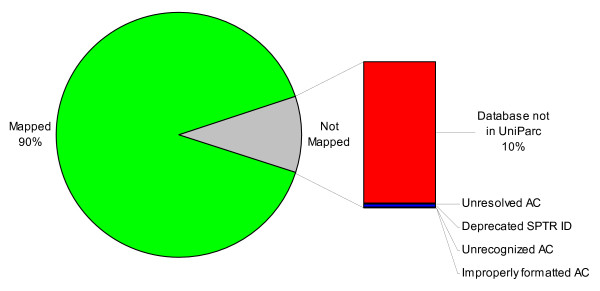
**Mapping of PRIDE identifications using PICR**. Of 339,696 identifications in PRIDE, 90% could be successfully mapped to one or more UPEntry entries. Of the remaining 10%, the vast majority originated from proprietary databases that did not provide the accompanying protein sequence information or from non-protein databases (gene or transcript identifiers). Less than 1% of the valid protein identifiers in PRIDE could not be mapped using PICR.

89% of PRIDE identifications come from 4 major data sources (IPI, Ensembl, NCBI and UniProtKB) but this still leaves 11% of identifications coming from secondary or proprietary databases. To test the coverage of PICR, we attempted to map the 339,696 current PRIDE identifications. The results of the mapping are shown in Figure 6.

90% of PRIDE identifications can be mapped to one or more UPEntry. Of the remaining 10% of identifications that are unmapped, less than 1% come from unresolved or badly formatted identifiers (including a large proportion of deprecated UniProt IDs, which are notoriously difficult to track once they are removed from circulation). The majority of the unmapped identifications originate from proprietary databases, for which the protein sequences have not been provided, or other databases not available in UniParc (mostly model organism gene and transcript identifiers). As such, most of the unmapped identifiers would have been difficult, if not impossible, to map with other available tools.

### Using the web interface

**Figure 7:**
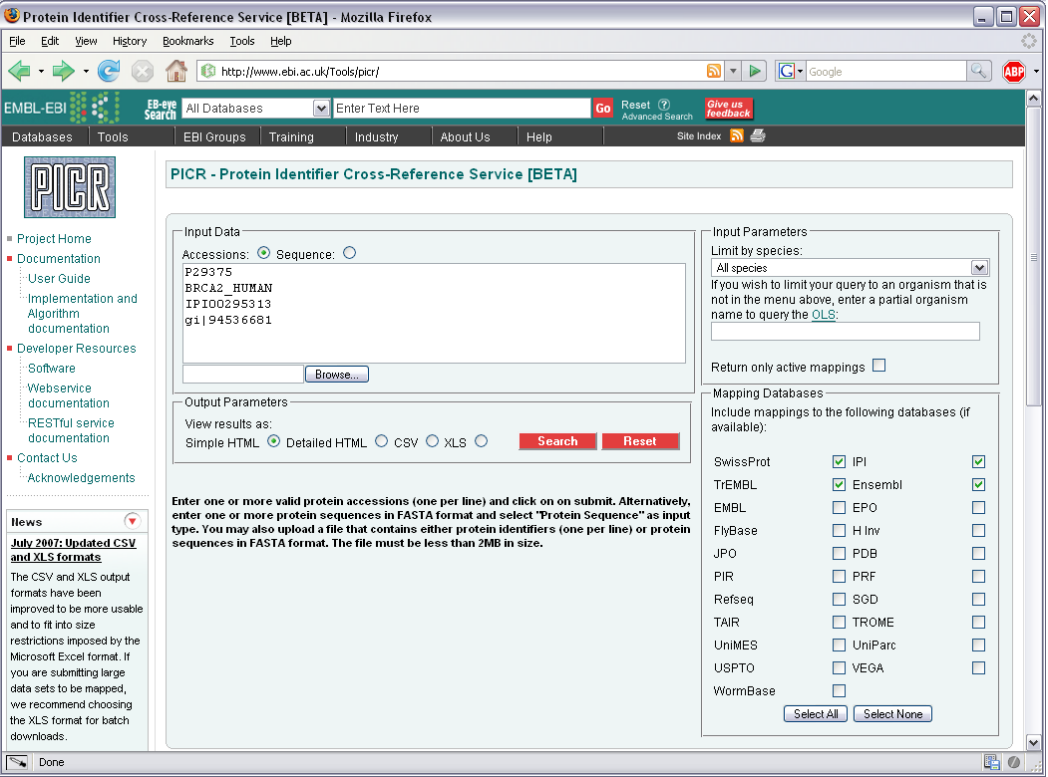
**PICR main search page**. The main search page of PICR, available online at [36].

Great care has been taken to design a user-friendly interface (Figure 7). The interface is divided into 4 sections. The first is for the *Input Data*, where the user can paste a list of protein identifiers in the text box, one identifier per line. Sequences in FASTA format can also be entered. Alternatively, users can click on the Browse button and select a text file to upload. If submitting sequences, the user must update the data type radio button to Sequences from Accessions.

Users can refine their search by changing values in the *Input Parameters *section. By default, PICR will only return active protein mappings across all species but it is possible to limit queries by taxonomy or expand them to include non-active mappings. To retrieve both active and non-active mappings, uncheck the 'Return only active mappings' box. To limit the mappings to a particular species, select the desired option from the 'Limit by species' menu. This menu contains the most common species present in UniParc, though over 140,000 distinct taxonomy ids are currently annotated in UniParc. If users wish to limit their searches to a species which is not predefined in the menu, they can type the organism name in the field provided.

**Figure 8:**
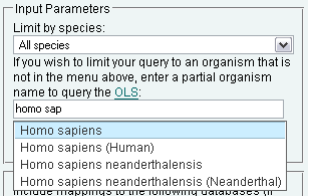
**Organism name auto-suggestion search**. PICR uses the OLS auto-complete AJAX interface components to provide source organism name lookups.

The web application will interactively query the Ontology Lookup Service [30] as the organism name is typed and will provide a list of suggested values (Figure 8).

If species are entered both in the selection menu and in the search box, the search box will take precedence. It must be noted that although we have tried to get the maximum taxonomical coverage for the mappings, some source databases do not provide taxonomy information and, as such, those mappings cannot be properly assigned to a taxon and will therefore be excluded from any search that is limited by taxonomy.

The next step involves selecting the databases the user wishes to map the input data to by updating the selections in the *Mapping Databases *section of the search form. To keep the interface light and simple, some mapping options actually refer to more than one database. For example, selecting Ensembl will query all the organism-specific Ensembl releases, as is the case for RefSeq, Vega [31] and Trome [32]. Selecting Swissprot and TREMBL will also include the respective splice variant databases [33].

**Figure 9:**
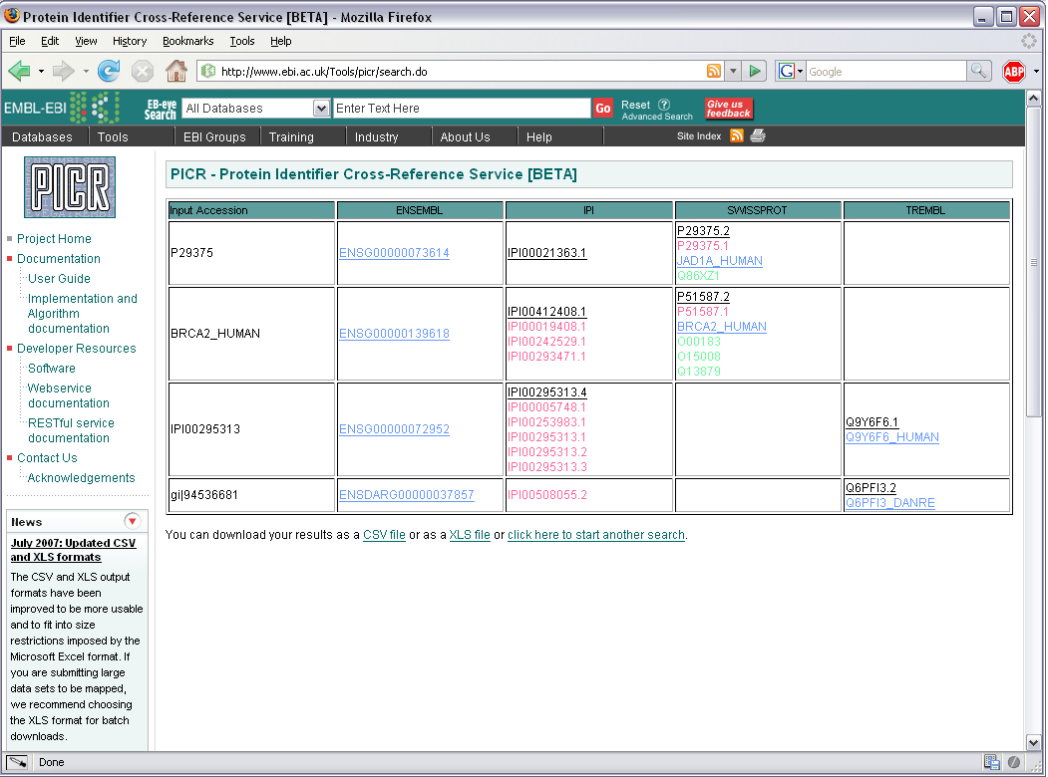
**Simple HTML view of search results**. A simple tabular HTML result display. Links go to the source databases where available. Mappings in red are inactive or deleted in the source   databases. Mappings in green are deprecated UniProt secondary identifiers. Links in blue come from UniProt data and are not guaranteed to be of 100% sequence identity with the submitted accession or sequence.

Finally, the user can choose how results should be presented. The default option is the 'Simple HTML' table view, where each row represents a submitted protein identifier or sequence and each column represents a selected mapping database (Figure 9). Some mappings might be highlighted in red. These mappings are historical and inactive, as the referenced entries have been removed or renamed from the current release of the mapped databases. Some mappings might be highlighted in green. These represent inactive, secondary UniProt identifiers. Some mappings might be highlighted in blue. These mappings, while valid, are the logical cross-references obtained from the mapping algorithm and may not be based on 100% sequence identity. All active mappings are hyperlinked to the original records from the source database if the user wishes to get more information on the entry.

**Figure 10:**
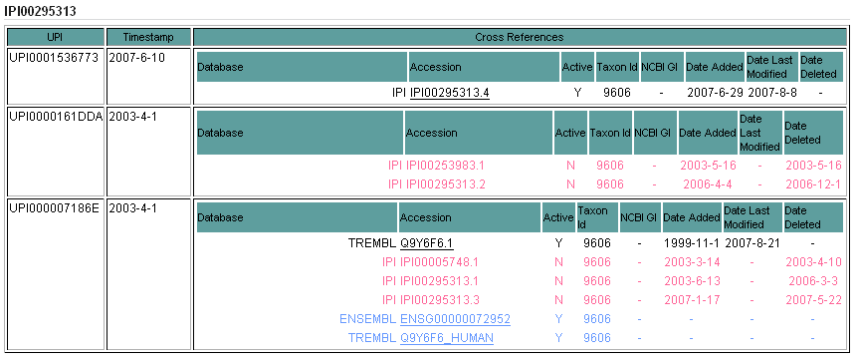
**Detailed HTML view of search results**. Partial view of a detailed HTML result display. Links go to the source databases where available. Mappings in red are inactive or deleted in the source   databases. Links in blue come from UniProt data and are not guaranteed to be of 100% sequence identity with the submitted accession or sequence. Additional data, such as timestamps, taxonomy source information and NCBI gis are displayed when available. It is possible to clearly observe the evolution of a protein identifier across multiple protein sequences.

The 'Detailed HTML' option will give a full description of each UniParc entry corresponding to the submitted protein accession or sequence, including the entry timestamp and a full description of the mappings (Figure 10).

**Figure 11:**
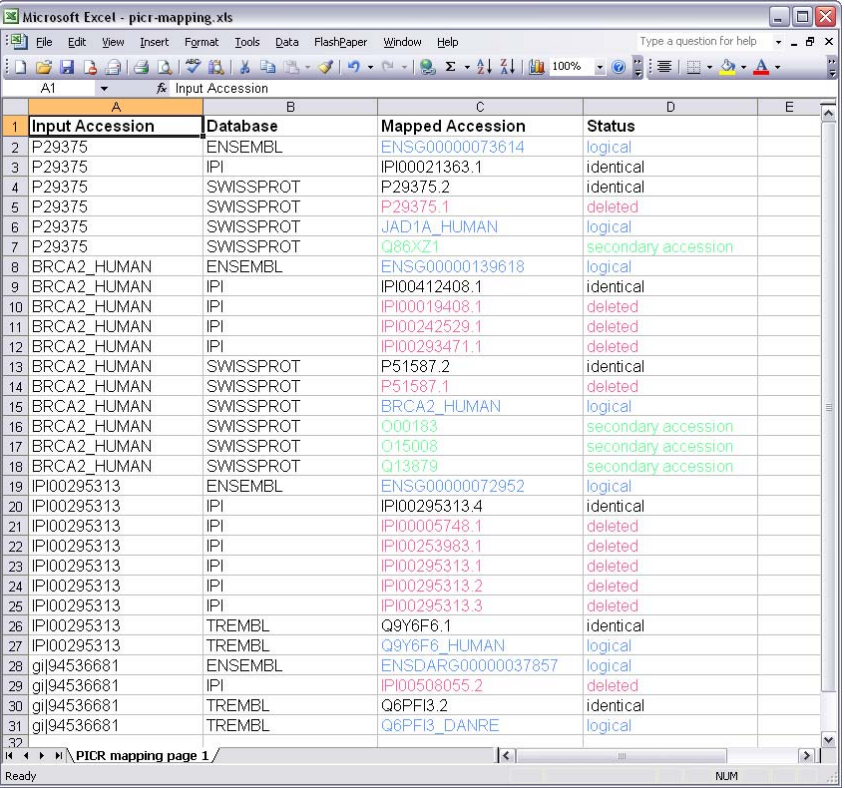
**XLS view of search results**. Search results can be downloaded in Microsoft Excel (XLS) or CSV format. Each line represents a mapping from a submitted identifier to one of the selected databases. The type of mapping (identical, logical, deleted or secondary accession) is also provided. The XLS format can retain colour-coding information provided in the web views. The CSV format cannot.

The 'XLS' option allows the download of the mappings as a tabulated Microsoft Excel file (Figure 11), with columns for the submitted identifier, mapping database, mapped accession and status. Each line represents one mapping from a submitted accession to a selected database and preserves the colour-coding information available in the web interface. The 'CSV' option allows the download of a comma-separated file with an identical layout to that of the Microsoft Excel file, though the colour-coding information is lost.

Generating the mappings is a computationally intensive process which may require calls to external services and can therefore take some time. To give the user interactive feedback on the status of the search in progress, a progress bar will be displayed on the screen as the search is processed and is updated, every second, using AJAX. When the search is complete, the results will be displayed on the screen or a file download dialog box will appear, depending on the selected options.

Users can submit any number of protein accessions or sequences to be mapped at a time. However, if more than 500 are submitted in one request, the user will be prompted to enter a valid email address and must select one of the file output formats (CSV or XLS). Once the search is done, an email is sent to the user providing a URL to download the generated result file.

### Using the SOAP and REST interfaces

PICR provides a publicly available SOAP web service to perform mappings. The service is encoded in the document/literal style for maximal interoperability. It is implemented in Java and deployed using JAX-WS to adhere with the latest WS-I specifications. Detailed developer documentation describing the SOAP service, as well as the WSDL descriptor file and sample Java client code examples are available online from the PICR website [34].

Representational State Transfer (REST) allows data elements to be associated with a well-formed URL. The same methods that are available in the SOAP interface are also available using the REST interface, with minor modifications to the parameters. Developer documentation on how to build valid REST queries is available online from the PICR website [35].

## Conclusion

Resolving protein identifiers from multiple data sources is a difficult problem and there was no existing solution generic enough to suit our needs. As such, we have created a powerful and flexible system that allows for the batch querying of protein identifiers and sequences against multiple data sources using the most comprehensive protein sequence data archive available.

Mappings can be limited by source database or taxonomic classification and the results can include data no longer available in source databases. This last feature is particularly useful when dealing with old data sets and literature citations.

We offer three distinct query interfaces: one interactive and two programmatic. The interactive web interface uses AJAX to enhance the browsing experience wherever possible and provides the possibility to obtain results in four different formats: simple HTML, detailed HTML, XLS and CSV. Users and application developers can query SOAP and REST interfaces programmatically to integrate PICR functionality in their applications or perform batch requests.

Our application will provide a valuable service to wide areas of the scientific community and plans are already underway to build on its success. Future work will include improving the gi number coverage with UniProt sequences. We are in communication with the NCBI to obtain daily up-to-date gi number to UniProtKB accession number mapping files, which will be incorporated into the UniParc data warehouse and made available via PICR. Furthermore, we plan to implement a similarity search to UniProt sequences. The mapping algorithm as presently available will be expanded such that users will be able to submit protein identifiers or sequences and obtain mappings to SwissProt and TREMBL based on a user-defined similarity threshold.

The application is freely available to use. Clients and code examples are available online under the Apache Open Source 2.0 License.

## Availability and requirements

• **Project name: **Protein Identifier Cross-Reference Service

• **Project home page: **

• **WSDL service descriptor: **

• **SOAP client demo: **

• **Operating system(s): **Platform independent

• **Programming language: **Java

• **Other requirements: **Java 1.5 or later, Apache Ant 1.6 or later

• **License: **Apache License 2.0

• Any restrictions to use by non-academics: none

## Abbreviations

AJAX- Asynchronous JavaScript and XML;

API- Application Programming Interface;

CRC- Cyclic Redundancy Check;

CSV- Comma-Separated Values;

NCBI- National Center for Biotechnology Information;

HTML- Hyper Text Mark-up Language;

PICR- Protein Identifier Cross-Referencing service;

REST- REpresentational State Transfer;

SOAP- Simple Object Access Protocol;

UniParc- Universal Protein database Archive;

UPI- UniParc Protein Identifier;

XML- Extensible Mark-up Language.

## Authors' contributions

RC, PJ and LM developed the mapping algorithm, based on original discussions with SK. RC implemented new UniParc data loaders and implemented the algorithm, the SOAP and REST interfaces as well as the interactive web interface. FR generated the SOAP stubs and bindings for the server-side code. QL and RL developed the new UniParc database schema extensions and are responsible for the UniParc production cycle. RA and HH developed the project concept. All authors read and approved the final manuscript.
